# Supplementary material for: Rhamnose biosynthesis is not impaired by the deletion of putative rfbC genes, slr0985 and slr1933, in Synechocystis sp. PCC 6803
Source: Appl Environ Microbiol. 2025 Jun 13;91(7):e00702-25. doi: 10.1128/aem.00702-25 (PMC12285223; doi:10.1128/aem.00702-25)
Supplement: Supplemental material — Tables S1 and S2; Figures S1 and S2. [file aem.00702-25-s0001.pdf]

Supplementary material

Rhamnose biosynthesis is not impaired by the deletion of putative *rfbC* genes, *slr0985* and *slr1933*, in *Synechocystis* sp. PCC 6803

João Pissarra[1,2,3], Marina Santos[1,2,3], Sara B. Pereira[1,2], Catarina C. Pacheco[1,2,3], Filipe Pinto[1,2,], Sónia S. Ferreira[5], Ricardo Monteiro[1,2], Cláudia Nunes[6], Manuel A. Coimbra[5], Didier Cabanes[1,2], Rita Mota[7], Paula Tamagnini[1,2,3] #

[1]i3S – Instituto de Investigação e Inovação em Saúde, Universidade do Porto, Porto, Portugal.

[2]IBMC – Instituto de Biologia Celular e Molecular, Universidade do Porto, Porto, Portugal.

[3]Instituto de Ciências Biomédicas Abel Salazar (ICBAS), Universidade do Porto, Programa Doutoral em Biologia Molecular e Celular (MCbiology), Porto, Portugal.

[4]Departamento de Biologia, Faculdade de Ciências, Universidade do Porto, Porto, Portugal.

[5]LAQV-REQUIMTE, Departamento de Química, Universidade de Aveiro, Aveiro, Portugal.

[6]CICECO – Instituto de Materiais de Aveiro, Departamento de Engenharia de Materiais e Cerâmica, Universidade de Aveiro, Aveiro, Portugal.

[7]acib GmbH - Austrian Centre of Industrial Biotechnology, Tulln, Austria.

# Address correspondence to Paula Tamagnini, pmtamagn@i3s.up.pt

Table S1 List of primers used for strain generation and confirmation.

Primer Name	Code	Primer Sequence 5' → 3'	Amplicon (bp)	Source
slr0985.5O[a,b]	1F	CGCCTGCGAAATCGTAGTAGCCAGTT ATC	709	This work
slr0985.5I[a]		GATAATTCGGTAATTGAGGCCTATGG GCTGACGGATAACGGTTCTGAGT		
slr0985.3O[a,b]	1R	GCTCATGGTAAACCGTATCAAATTGG GTC	1157	
slr0985.3I[a]		TTATCCGTCAGCCCATAGGCCTCAAT TACCGAATTATCCCAGCGAGACC		
slr0985.5F[b]	2F	TCGCCATTCAGGCTGCGCAACGGAG ATTGGTCAGAGGATCGTCTCATTCC	535	
slr0985.5R[b]	2R	TAGGCGAGGACCCGGGTCAGATAGC AAGGTCTGACAGAGC		
slr0985_SB_Fwd[c]	3F	CCTGATGTTGTCCGCGCCCTA	333	
slr0985_SB_Rev[c]	3R	CCAACTAGGACGATATCCCAGTA		
slr1933.3F[a,c]	6F	TTTAATCTCCCGGGGTTCGCCTCTTTC CTGAATTACCCAAAAGTTTGC	574	
slr1933.3R[a,c]	6R	CACTCATTAGGCACCCCAGGCCCACA ATTTTGCGTTGGTTCCTGG		
slr1933.5F[a]		TCGCCATTCAGGCTGCGCAACCCATG GCTGGAAATAACCATAGACCGTTCG	680	
slr1933.5R[a]		GGCGAACCCCGGGAGATTAAATTCCT CTTTAGGGTTACGACACC		
slr1933.FwdO[b]	4F	CTTTGAGCCGAACTTTCTC	1836 (WT) 2080 (MT)	
slr1933.RevO[b]	4R	CATACCGTTGAACAATGGAC		
slr1933.FwdI[b]	5F	CTTTGAGAGCTACAACGACAAG	347	
slr1933.RevI[b]	5R	CATAGGACGGTGCATAGTAATC		
pSEVA351.Cm.Fwd[d]		GCGAAACCCGGGCGTTGATCGGCACG TAAGAG	805	
pSEVA351.Cm.Rev[d]		TCCACACCCGGGACGATTTAAGTCAAA TTACGCCC		

[a]Primers used for plasmids construction.
[b]Primers used for the confirmation of the knockout strains' segregation by PCR.
[c]Primers used for Southern blot probes.
[d]Primers used for the amplification of the *cat* cassette.

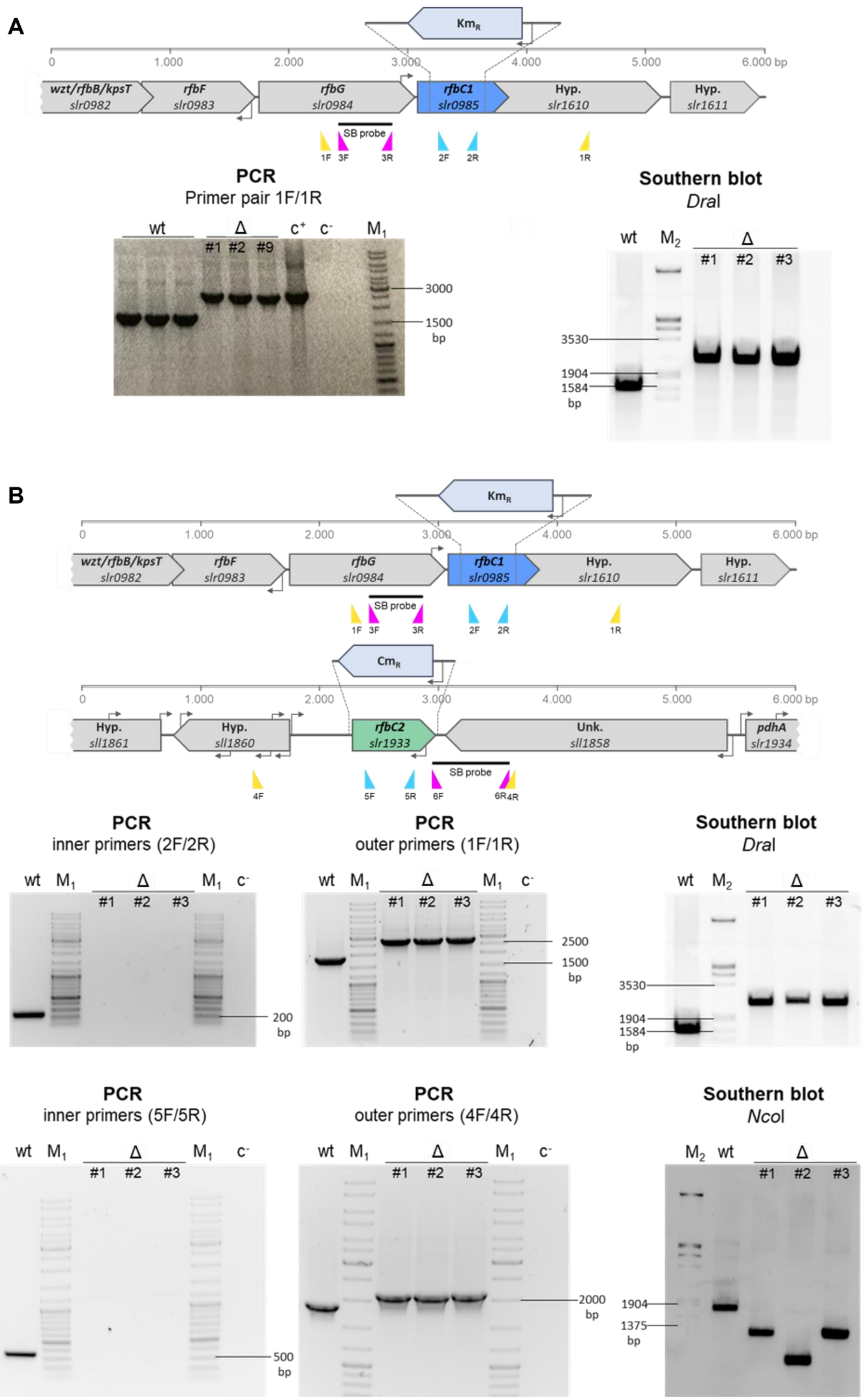
A
KmR
0
1.000
2.000
3.000
4.000
5.000
6.000 bp
wzt/rfbB/kpsT
slr0982
rfbF
slr0983
rfbG
slr0984
rfbC1
slr0985
Hyp.
slr1610
Hyp.
slr1611
SB probe
1F
3F
3R
2F
2R
1R
PCR
Primer pair 1F/1R
wt
Δ
#1
#2
#9
c+
c-
M1
3000
1500
bp
Southern blot
DraI
wt
M2
Δ
#1
#2
#3
3530
1904
1584
bp
B
KmR
0
1.000
2.000
3.000
4.000
5.000
6.000 bp
wzt/rfbB/kpsT
slr0982
rfbF
slr0983
rfbG
slr0984
rfbC1
slr0985
Hyp.
slr1610
Hyp.
slr1611
SB probe
1F
3F
3R
2F
2R
1R
CmR
0
1.000
2.000
3.000
4.000
5.000
6.000 bp
Hyp.
sll1861
Hyp.
sll1860
rfbC2
slr1933
Unk.
sll1858
pdhA
slr1934
SB probe
4F
5F
5R
6F
6R
4R
PCR
inner primers (2F/2R)
wt
M1
Δ
#1
#2
#3
M1
c-
200
bp
PCR
outer primers (1F/1R)
wt
M1
Δ
#1
#2
#3
M1
c-
2500
1500
bp
Southern blot
DraI
wt
M2
Δ
#1
#2
#3
3530
1904
1584
bp
PCR
inner primers (5F/5R)
wt
M1
Δ
#1
#2
#3
M1
c-
500
bp
PCR
outer primers (4F/4R)
wt
M1
Δ
#1
#2
#3
M1
c-
2000
bp
Southern blot
NcoI
M2
wt
Δ
#1
#2
#3
1904
1375
bp

Fig S1 RfbC encoding genes in *Synechocystis* sp. PCC 6803, and confirmation of the segregation of the Δ*rfbC1* and Δ*rfbC1*Δ*rfbC2* strains. **(A)** Schematic representation of the genomic context, position of the primers used in the PCR reactions, and regions replaced by the selection cassette (conferring resistance to kanamycin). Agarose gel electrophoresis showing the PCR amplicons obtained with a primer pair targeting the *rfbC1* region (slr0985.5O/slr0985.3O, expected sizes 1828 bp for the wild type and 2778 bp for the knockout strain). Southern blot hybridization with genomic DNA from *Synechocystis* wild type and Δ*rfbC1* clones digested with *Dra*I and hybridized with a probe covering the 5' flanking region of *rfbC1* gene (obtained by PCR with the primer pair slr0985_SB_Fwd/slr0985_SB_Rev). Expected sizes of restriction fragments are 1636 bp for wild type and 2586 bp for the knockout strain clones. **(B)** Schematic representation of the genomic context, position of the primers used in the PCR reactions, and regions replaced by the selection cassettes (conferring resistance to kanamycin or chloramphenicol). Agarose gel electrophoresis showing the PCR amplicons obtained with a primer pair targeting *rfbC1* (inner primers: slr0985.F/slr0985.R, expected size 232 bp for the wild type) and the gene region (outer primers: slr0985.5O/slr0985.3O, expected sizes 1828 bp for the wild type and 2778 bp for the knockout strain). Southern blot hybridization with genomic DNA from *Synechocystis* wild type and Δ*rfbC1*Δ*rfbC2* clones digested with *Dra*I and hybridized with a probe covering the 5' flanking region of *rfbC1* gene (obtained by PCR with the primer pair slr0985_SB_Fwd / slr0985_SB_Rev). Expected sizes of restriction fragments are 1636 bp for wild type and 2586 bp for the knockout strain clones. Agarose gel electrophoresis showing the PCR amplicons obtained with a primer pair targeting *rfbC2* (inner primers: slr1933.FwdI/slr1933.RevI, expected size 347 bp for the wild type) and the gene region (outer primers: slr1933.FwdO/slr1933.RevO, expected sizes 1836 bp for the wild type and 2080 bp for the knockout strain). Southern blot hybridization with genomic DNA from *Synechocystis* wild type and Δ*rfbC1*Δ*rfbC2* clones digested with *Nco*I and hybridized with a probe covering the 3' flanking region of *rfbC2* gene (obtained by PCR with the primer pair slr1933.3F/slr1933.3R). Expected sizes of restriction fragments are 1819 bp for wild type and 1256 or 795 bp for the knockout strain clones, depending on the orientation of the resistance cassette.

wt - wild type, Δ - knockout strain, # clone number, c^+ - positive control, c^- - negative control, M1 - GeneRuler DNA Ladder mix (Thermo Scientific), M2 – DNA Weight Marker III DIG-Labeled (Roche).

Table S2 List of primers used in the RT-qPCR analysis.

Primer Name	Primer Sequence 5' → 3'	Amplicon (bp)	Source
sll1395_RT_fwd	GAAATCGGTGCCTATGTG	196	This work
sll1395_RT_rev	CATGGACTCCATAAACCC		
slr0982_RT_fwd	ATTGATGAGCCCCTAACG	217	
slr0982_RT_rev	ATCCCACCGAGTAAACAC		
slr0985_RT_fwd	CTGAGGAGTTGAAGGAAG	181	
slr0985_RT_rev	GAATCAACCCGCAAATCC		
slr1933_RT_fwd	GGTTGTGTGCTCAATGAC	130	
slr1933_RT_rev	CATAGGACGGTGCATAGT		
slr1072_RT_fwd_ii	CAGGTGGTTTCACAGGTCAA	218	
slr1072_RT_rev_ii	TCATCAGCGGCGGAATTG		
sll1213_RT_fwd_ii	CTGGAATCCTACCGCTTGC	215	
sll1213_RT_rev_ii	GCCGCATCAGTGGAGTAAA		
sll1212_RT_fwd_ii	CGGCATCCAAGTAAGGTTC	181	
sll1212_RT_rev_ii	GCCGTTACAGGCGAATAAGTC		
slr0983_RT_fwd_ii	GCTACGGTCACAGCAGTTG	196	
slr0983_RT_rev_ii	ATTCAGTTAGGGGCTCACC		
slr1610_RT_fwd_ii	GCCCAGACCCAATTTGAT	189	
slr1610_RT_rev_ii	TTCCCTTACTCGCTCACT		

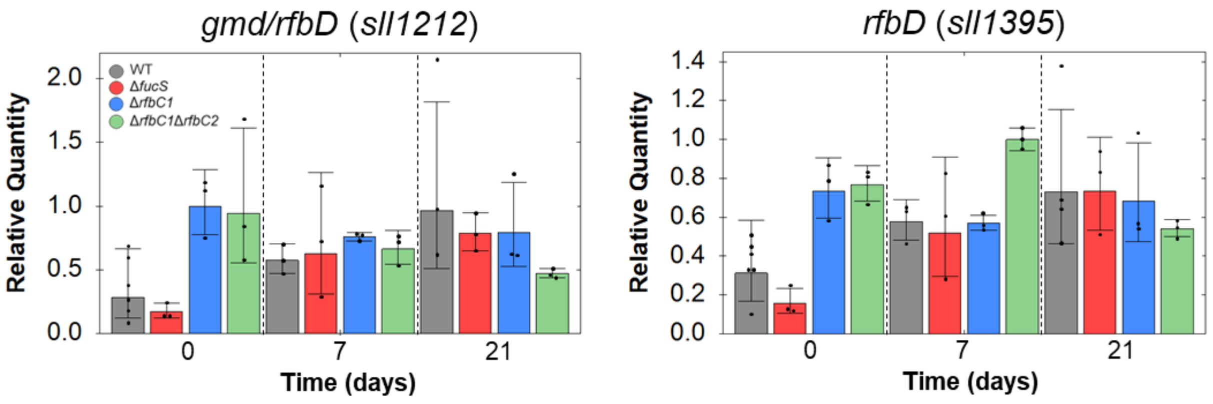

Fig S2 RT-qPCR analysis of *sll1212* and *sll1395* relative expression in *Synechocystis* sp. PCC 6803 wild type (WT) and Δ*fucS*, Δ*rfbC1* and Δ*rfbC1*Δ*rfbC2* strains. RNA was extracted from cells of the different strains collected at three time points - 0, 7, and 21 days. The bars represent the expression of *gmd/rfbD* (*sll1212*) and *rfbD* (*sll1395*) from *Synechocystis* sp. PCC 6803 wild type, Δ*fucS*, Δ*rfbC1* and Δ*rfbC1*Δ*rfbC2* at each time point. The data were obtained from three biological replicates with technical triplicates, error bars represent the standard deviations, and individual measurements are shown.
